# Supplementary material for: Signal theory based encryption of faster-than-Nyquist signals for fiber and wireless transmission
Source: Commun Eng. 2025 Jan 29;4:13. doi: 10.1038/s44172-025-00351-3 (PMC11779797; doi:10.1038/s44172-025-00351-3)
Supplement: Supplementary file 2 — Supplementary material [file 44172_2025_351_MOESM2_ESM.pdf]

Signal Theory Based Encryption of Faster-than-Nyquist Signals for Fiber and Wireless Transmission: Supplementary material

ABHINAND VENUGOPALAN[1,*], KARANVEER SINGH[1], JANOSCH MEIER[1], AND THOMAS SCHNEIDER[1]

This document provides supplementary information to "Signal Theory Based Encryption of Faster-than-Nyquist Signals for Fiber and Wireless Transmission". It contains the theory of encryption and simulation results for the calculation of the encryption strength.

Supplementary note 1:

Theory

According to the sampling theorem, a signal of baseband bandwidth $B_i/2$, can be written as:

$$s_i(t) = \sum_{m=-\infty}^{\infty} s_i\left(\frac{m}{B_i}\right) \cdot \mathrm{sinc}(B_i t - m) \ . \tag{1}$$

Therefore, the signal $s_i(t)$ is completely described as a superposition of time-shifted sinc pulses (sinc) of bandwidth B_i, each of which weighted with a single sampling value $s_i(m/B_i)$. However, Eq. (1) is a mathematical construct and cannot be achieved practically as sinc-pulses are unlimited in the time-domain. Alternatively, the same signal $s_i(t)$ can be described by a superposition of sinc-pulse sequences (SPS) [1] defined by:

$$\lim_{\substack{x \to t \\ \frac{B_i x}{N_i} \in \mathbb{R}\setminus\mathbb{Z}}} \frac{\sin(\pi B_i x)}{N_i \sin\left(\frac{\pi B_i x}{N_i}\right)} = \mathrm{sq}_{N_i,B_i}(t) = \frac{2}{N_i}\left(\frac{1}{2} + \sum_{l=1}^{\frac{N_i-1}{2}} \cos\left(\frac{2\pi l B_i t}{N_i}\right)\right) . \tag{2}$$

According to Eq. (2), such an SPS corresponds to a flat, rectangular, phase-locked frequency comb. The SPS has the same bandwidth as the single sinc pulse $B_i = N_i \Delta f$ but, it is periodical with the repetition rate Δf and has $N_i - 1$ zero crossings with an interval of $1/B_i$ from the pulse peak. Identical SPS, time-shifted to these zero crossings are orthogonal to each other. As for the single sinc pulse, this orthogonality allows the inter-symbol-interference-free representation of the signal [1].

Setting the individual phases of the spectral lines in the frequency comb to an arbitrary value, the sequence can be described by:

$$\mathrm{sqG}_{N_i,B_i}(t) = \frac{2}{N_i}\left(\frac{1}{2} + \sum_{l=1}^{\frac{N_i-1}{2}} \cos\left(\frac{2\pi l B_i t}{N_i} + \phi_l\right)\right). \tag{3}$$

[1] THz-Photonics Group, Technische Universität Braunschweig, 38106 Braunschweig, Germany.

[*] Email: abhinand.venugopalan@tu-braunschweig.de

This process, however, removes the orthogonality between time shifted copies of the SPS. The multiplication of a sub-signal with this pulse sequence results in a noise-like sub-signal, and to recover the original sub-signal the exact same pulse sequence will be required at the receiver. Therefore, the pulse sequence sqG_{N_i,B_i} is the *keyfunction,* which can be used to encrypt and decrypt any sub-signal of bandwidth B_i/N_i.

For encryption, the signal $s_i(t)$ of bandwidth B_i is divided into N_i parallel branches to generate low bandwidth sub-signals of bandwidth B_i/N_i. In each of these parallel branches, a time delayed copy of the keyfunction is multiplied with the sub-signal. The time delay between the branches is $t_{l,i} = (l-1)/B_i$, where l is the lth branch and varies from 1 to N_i. Afterwards, all N_i sub-signals are added up to generate the encrypted signal. The encrypted signal $s_i'(t)$ can be described by:

$$s_i'(t) = \overbrace{\sum_{l=1}^{N_i} \underbrace{\sum_{p=-\infty}^{\infty} s_i\left(\frac{l-1}{B_i} + \frac{N_i p}{B_i}\right) \cdot \mathrm{sinc}\left(\frac{B_i}{N_i} t + \frac{l-1}{N_i} - p\right)}_{sub-signal\ in\ the\ l^{th}-parallel\ branch} \cdot \underbrace{\mathrm{sqG}_{N_i,B_i}\left(t - \frac{l-1}{B_i}\right)}_{\substack{keyfunction \\ time\ delayed\ by\ \frac{l-1}{B_i}}}}^{summation\ of\ all\ l\ encrypted\ sub-signals}. \tag{4}$$

This encrypted signal has the same bandwidth B_i as the original one and a number of k such signals can form an encrypted super-signal of bandwidth $B_{\mathrm{SE}} = \sum_{i=1}^{k} B_i$.
The decryption of the signal $s_i'(t)$ is based on the orthogonality, that results after the baseband rectangular filter $\Pi(N_i f/B_i)$ in the detector:

$$\left[\mathbf{F}_f^{-1}\left(\left[\mathbf{F}_{t'}\left(\mathrm{sqG}_{N_i,B_i}\left(t' - \frac{m}{B_i}\right) \cdot \mathrm{sqG}_{N_i,B_i}\left(t' - \frac{q}{B_i}\right)\right)\right](f) \cdot \Pi\left(\frac{N_i f}{B_i}\right)\right)\right](t) = \begin{cases} \frac{1}{N_i}, & m - q \in N_i\mathbb{Z} \\ 0, & \text{else} \end{cases}. \tag{5}$$

Here m and q are integers and $\mathbf{F}$, $\mathbf{F}^{-1}$ symbolize the forward and inverse Fourier transform, respectively. The rectangular filter function is defined by $\Pi(f)$ equals 1 for $|f| < \frac{1}{2}, \frac{1}{2}$ for $|f| = \frac{1}{2}$ and 0 elsewhere. A detailed mathematical derivation of Eq. (5) can be found in the section (Filter Orthogonality) of this supplement. Please note, that for making use of this orthogonality not only the keyfunction but, as well the bandwidth and center frequency of the filter has to be known.
The information extraction with the same keyfunction can be described by:

$$\begin{aligned} &\left[\mathbf{F}_f^{-1}\left(\left[\mathbf{F}_{t'}\left(s_i'(t) \cdot \mathrm{sqG}_{N_i,B_i}\left(t' - \frac{q}{B_i}\right)\right)\right](f) \cdot \Pi\left(\frac{N_i f}{B_i}\right)\right)\right](t) \\ &= \left[\mathbf{F}_f^{-1}\left(\left[\mathbf{F}_{t'}\left(\sum_{p=-\infty}^{\infty} s_i\left(\frac{q}{B_i} + \frac{N_i p}{B_i}\right) \cdot \mathrm{sinc}\left(\frac{B_i}{N_i} t' + \frac{q}{N_i} - p\right) \cdot \mathrm{sqG}_{N_i,B_i}\left(t' - \frac{q}{B_i}\right) \cdot \mathrm{sqG}_{N_i,B_i}\left(t' - \frac{q}{B_i}\right)\right)\right](f) \cdot \Pi\left(\frac{N_i f}{B_i}\right)\right)\right](t) \\ &= \frac{1}{N_i} \sum_{p=-\infty}^{\infty} s_i\left(\frac{q}{B_i} + \frac{N_i p}{B_i}\right) \cdot \mathrm{sinc}\left(\frac{B_i}{N_i} t + \frac{q}{N_i} - p\right). \end{aligned} \tag{6}$$

In contrast to Eq. (5), here q is the limited time shift of a single interval from 0 to $N_i - 1$. So, apart from the constant factor $1/N_i$, Eq. (6) corresponds to the decrypted sub-signal in the $l - 1 = q$-th branch as defined in Eq. (4).

Supplementary note 2:

Simulation analysis

The security strength of the encryption has been analyzed by simulations with the Optisystem software package.

Single signal encryption:
To test the encryption, a 4-QAM, 90 GBd pseudo random bit sequence (PRBS) in its Nyquist bandwidth of B = 90 GHz was encrypted with an N = 9 line keyfunction. Therefore, as described in Fig. 2 of the main paper, the sampling points were first

divided between the 9 branches and in each branch these sub-samples were used to generate a 10 GHz sub-signal. These sub-signals are multiplied with time shifted ($1/B_i$ between the branches) copies of the keyfunction and added up to generate the 90 GBd encrypted signal. Afterwards, the encrypted signal is modulated on a 193.4 THz carrier (in the conventional (C) band of telecommunications).
In the receiver, the encrypted signal is power-split into 9-branches and subjected to parallel coherent detection. The multiplication with the keyfunction was realized at the coherent detector, as described in Fig. 5 of the main paper. Therefore, the time-shifted keyfunction was modulated to the local oscillator and in each branch the decrypted sub-signal can be detected, if the bandwidth of the coherent detector is set to B_i/N_i.
The constellation diagram of one of these sub-signals after detection is shown in Fig. 1. When a wrong keyfunction is used, the decryption of the PRBS signal fails and the constellation points are dispersed in the IQ-diagram, as depicted in Fig. 1 (a).
Even if the eavesdropper has access to the transmission and is able to define the transmitted signal, no information about the keyfunction is revealed by the measurement, as can be seen for the transmission of a continuous amplitude (just ones) in Fig. 1 (b) and alternating ones and zeros in Fig. 1 (c). Without knowledge of the keyfunction, only a noise-like signal without any information about the signal or the keyfunction can be measured. When the keyfunction is known, the original PRBS signal is recovered and the measured constellation diagram of the PRBS is shown in Fig. 1 (d).

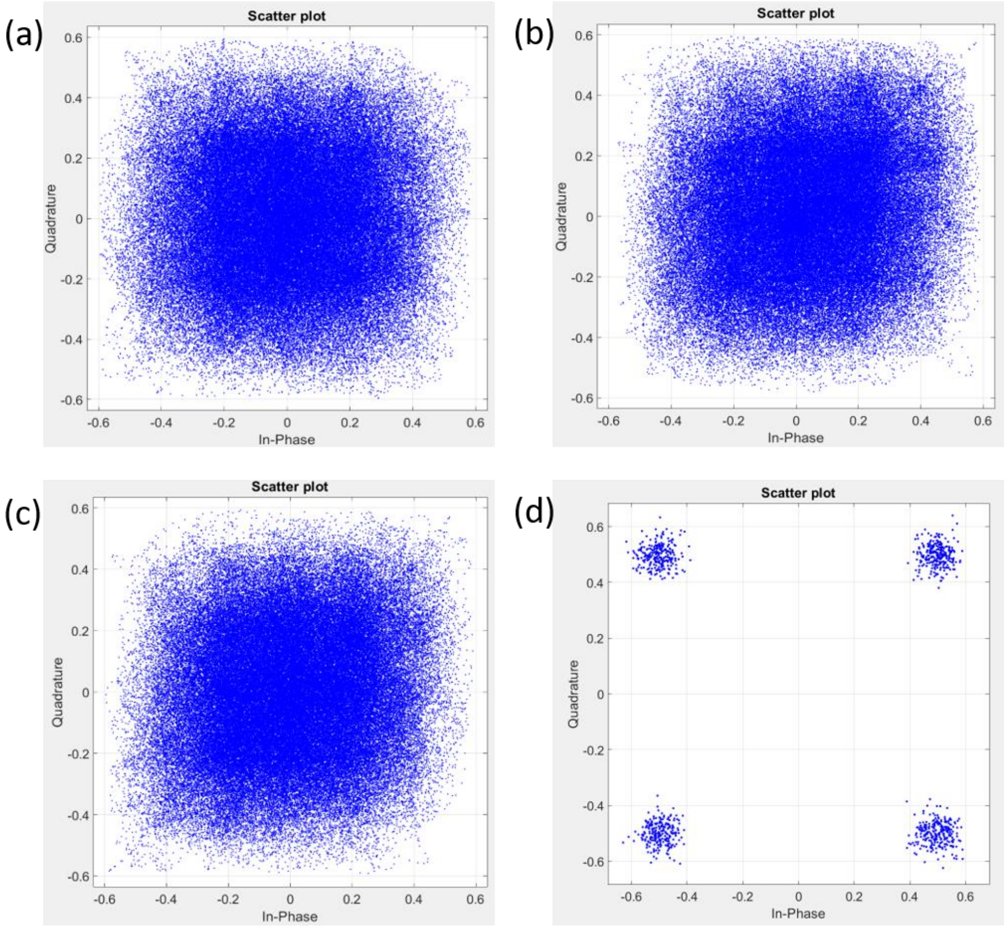

Fig. 1 Constellation diagram after signal detection when a wrong keyfunction is used at the receiver (a, b, c). A pseudo random bit sequence (PRBS-7) is shown in (a), a continuous amplitude (just ones) and alternating ones and zeros were transmitted in (b) and (c), respectively. The PRBS-7 constellation diagram for the matching keyfunction is shown in (d).

Key space:
To estimate the security strength for the encryption of a single signal, the number of possible keys for a given N is evaluated. Since the phase of the comb lines defines a part of the keyfunction, we have simulated the possible deviation from these phase values $\Delta\phi$ that still allows a decryption of the signal with a bit error rate lower than 10^{-3}, as defined by the FEC limit for forward error correction [2]. Then, the key strength can be calculated from this as $key_1(\Delta\phi, n) = \text{floor}\left(\frac{360^0}{\Delta\phi}\right)^n$. Since for the decryption not only the key but as well the center frequency and bandwidth of the signal are required, we have called this key_1.

For the N = (2n+1) line frequency comb, the deviation is investigated by varying one of the phase components from its ideal position while fixing the remaining $n-1$ components as defined by the keyfunction. The same process is repeated for all n lines and averaged. The result for N = 9, 15, 25 and 45 lines and a signal-to-noise ratio (SNR) of 15 dB is plotted in Fig. 2 (a). As can be seen, the possible phase variation for the single line is almost constant for all these numbers of lines with a $\Delta\phi$ of around 70°. When one of the lines is out of phase, the possible range for a second line decreases, as shown in Fig. 2 (b) for N = 25 and again an SNR of 15 dB. As presented, the sum of the phases is around 60°, which corresponds to (a). The key as well depends on the noise level, as shown in Fig. 2 (c) for N = 25.

With these results, the key strength can be estimated from Fig. 2 (d) for an SNR of 15 dB and $\Delta\phi$ = 70°. As long as the sampling theorem is not violated, N can be any odd number. So, with N = 201, for instance, around 10^{70} phase variations of the key are possible for an SNR of 15 dB.

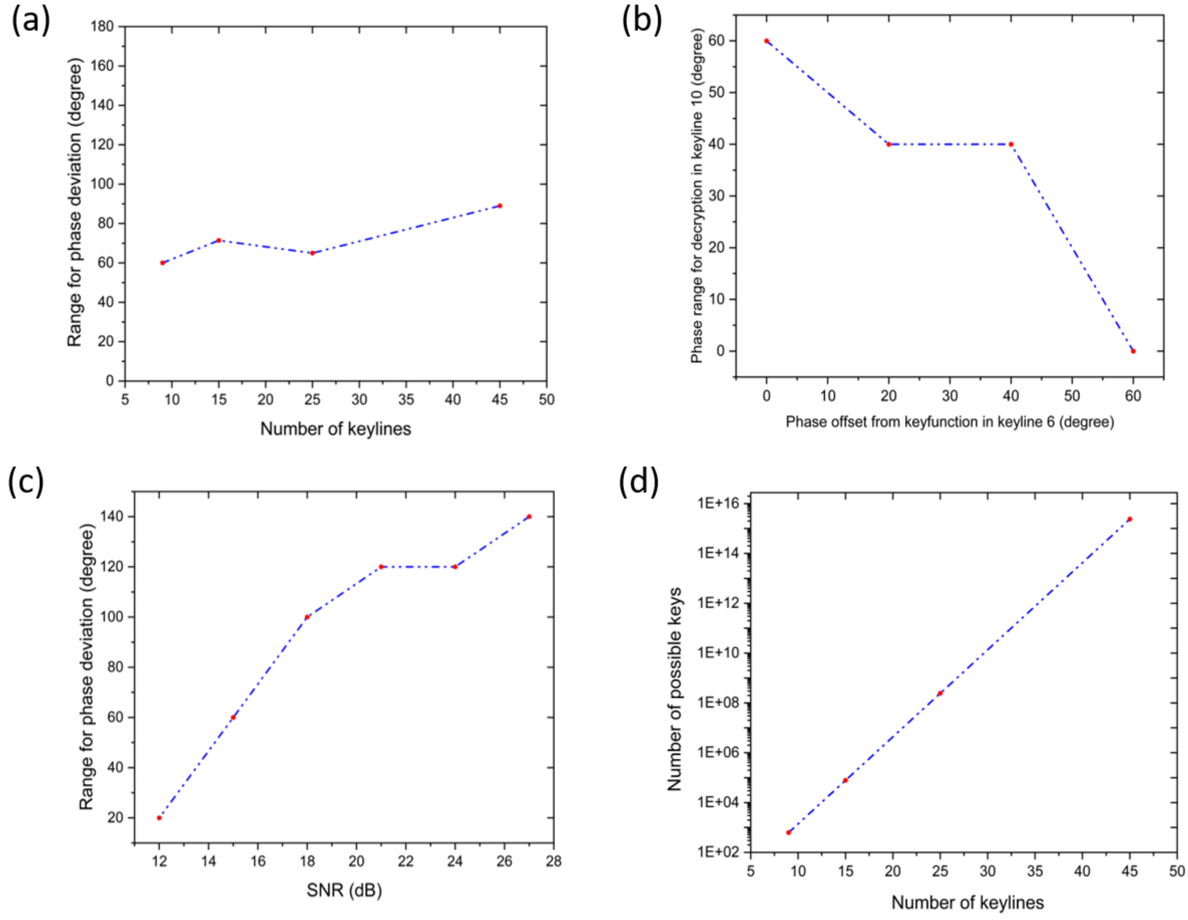

Fig. 2 Simulation results for the encryption strength based on the keyfunction. If just one line of the keyfunction has a different phase than defined by the key, the possible phase range in which the signal can still be decrypted is shown in (a) for an SNR of 15 dB. In (b) two phases of the 25-line keyfunction are different to the correct key and the possible phase difference of the second line in dependence on the phase difference of the first is shown for an SNR of 15 dB. The SNR dependence for a 25-line keyfunction is shown in (c). The corresponding number of possible keys for a given bandwidth at an SNR of 15 dB and a $\Delta\phi$ of 70° is shown in (d).

Encryption in a super-signal:

For the simulation we assumed a three-line frequency comb with 90 GHz spacing (193.31 THz, 193.40 THz and 193.49 THz) as three carriers for the super-signal generation. On each of the three carriers a 90 GBd BPSK signal was encrypted to form the 270 GBd super-signal in a bandwidth of 270 GHz.

For detecting one of the 90 GBd signals, the 270 GHz super-signal is multiplied with the correct keyfunction modulated on the LO wave in the coherent detector. To measure how accurately the bandwidth of the signal within the 270 GHz super-signal has to be known, a sweep for the signal bandwidth expected at the receiver is carried out. The results can be seen in Fig. 3. So, already a very small deviation of the bandwidth will destroy the possible detection of the signal. And the very small range in which a detection is possible decreases with the number of keylines N.

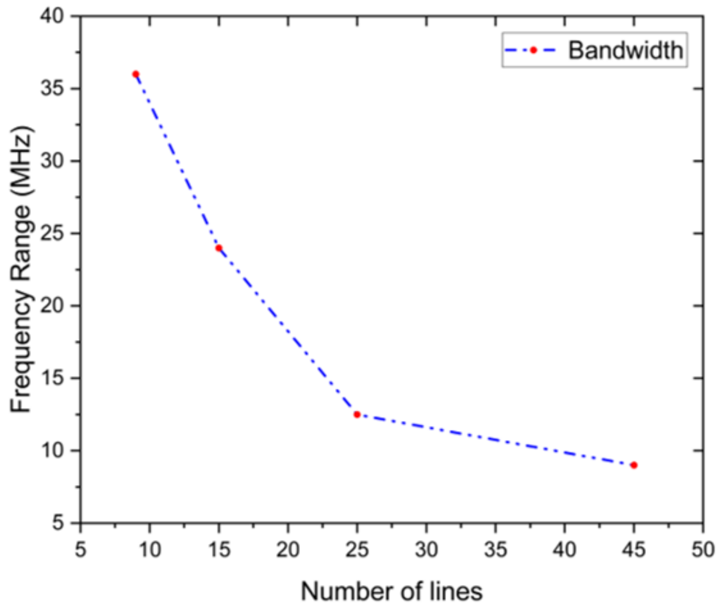

Fig. 3 Minimum accuracy required for the bandwidth for detecting a 90 GHz signal from an encrypted super-signal of bandwidth 270 GHz, if the keyfunction is known.

An eavesdropper with knowledge of the keyfunction still has to locate the center frequency and the signal bandwidth within the super-signal. Since the bandwidth of the encrypted signal exceeds that of state-of-the art detectors, memories and signal processing devices, the eavesdropper may first filter slices of the super-signal by optical filters and store it for post-processing in a brute force attack. If the signal is within the slices and not already clipped by the filter, the eavesdropper may generate the correct keyfunction of different bandwidth, and apply it to the slices with an assumed central frequency. Therefore, $key_2(\Delta f_c, B_{SE}) = \mathrm{floor}\left(\frac{B_{SE}}{\Delta f_c}\right)$ gives the number of possibilities that have to be considered by the eavesdropper for the signal center to be within the bandwidth of the super-signal B_{SE}. Here, Δf_c is the possible frequency offset for the center frequency in which a decryption is still possible. In an analog manner, $key_3(\Delta f_b, f_l) = \mathrm{floor}\left(\frac{f_l}{\Delta f_b}\right)$ corresponds to the possibilities for the bandwidth mismatch which must be considered by an attacker, with f_l as the possible bandwidth range in which the attacker assumes the signal. Here, Δf_b, which is shown in Fig. 3, is the bandwidth mismatch for which a decryption is still possible. For normal DWDM communication signals, the maximum bandwidth can be up to 100 GHz.

The total number of possibilities is given by the product $key_1(\Delta\phi, n) \cdot key_2(\Delta f_c, B_{SE}) \cdot key_3(\Delta f_b, f_l)$. Following our simulations for 25 lines and 15 dB SNR, we calculated the number of different keyfunctions to be considered by an eavesdropper to be around 10^{16} ($\Delta\phi = 70^o$, $\Delta f_c = 100$ MHz, $B_{SE} = 1$ THz, $\Delta f_b = 0.0125$ GHz, $f_l = 87.5$ GHz). Thereby, to specify f_l, the considered comb bandwidth was ranging from 12.5 GHz to 100 GHz. With extrapolating our simulations to $N = 201$ lines, we get an estimated key space of around 10^{77}.

Filter Orthogonality

To show that Eq. (5) is true, we expand it:

$$\left[\mathbf{F}_f^{-1}\left(\left[\mathbf{F}_{t'}\left(\mathrm{sqG}_{N_i,B_i}\left(t'-\frac{m}{B_i}\right)\cdot\mathrm{sqG}_{N_i,B_i}\left(t'-\frac{q}{B_i}\right)\right)\right](f)\cdot\Pi\left(\frac{N_i f}{B_i}\right)\right)\right](t)$$

First, $\overset{(1)}{=}$ by inserting the definition of the keyfunction from Eq. (3):

$$\overset{(1)}{=}\left[\mathbf{F}_f^{-1}\left(\left[\mathbf{F}_{t'}\left(\left(\frac{1}{N_i}+\sum_{l=1}^{\frac{N_i-1}{2}}\frac{2}{N_i}\cos\left(\frac{2\pi l B_i t'}{N_i}-\frac{2\pi l m}{N_i}+\phi_l\right)\right)\cdot\left(\frac{1}{N_i}+\sum_{l=1}^{\frac{N_i-1}{2}}\frac{2}{N_i}\cos\left(\frac{2\pi l B_i t'}{N_i}-\frac{2\pi l q}{N_i}+\phi_l\right)\right)\right)\right](f)\cdot\Pi\left(\frac{N_i f}{B_i}\right)\right)\right](t)$$

This can be re-written to $\overset{(2)}{=}$:

$$\overset{(2)}{=}\left[\mathbf{F}_f^{-1}\left(\left[\mathbf{F}_{t'}\left(\left(\sum_{l=1}^{\frac{N_i-1}{2}}\frac{2}{N_i}\cos\left(\frac{2\pi lB_it'}{N_i}-\frac{2\pi lm}{N_i}+\phi_l\right)\right)\cdot\left(\sum_{l=1}^{\frac{N_i-1}{2}}\frac{2}{N_i}\cos\left(\frac{2\pi lB_it'}{N_i}-\frac{2\pi lq}{N_i}+\phi_l\right)\right)\right.\right.\right.\right.$$
$$\left.\left.\left.\left.+\sum_{l=1}^{\frac{N_i-1}{2}}\frac{2}{N_i^2}\cos\left(\frac{2\pi lB_it'}{N_i}-\frac{2\pi lm}{N_i}+\phi_l\right)+\sum_{l=1}^{\frac{N_i-1}{2}}\frac{2}{N_i^2}\cos\left(\frac{2\pi lB_it'}{N_i}-\frac{2\pi lq}{N_i}+\phi_l\right)+\frac{1}{N_i^2}\right)\right](f)\cdot\Pi\left(\frac{N_if}{B_i}\right)\right)\right](t)$$

by rewriting the sum product to a double sum it follows $\overset{(3)}{=}$:

$$\overset{(3)}{=}\left[\mathbf{F}_f^{-1}\left(\left[\mathbf{F}_{t'}\left(\sum_{v=1}^{\frac{N_i-1}{2}}\sum_{l=1}^{\frac{N_i-1}{2}}\frac{4}{N_i^2}\cos\left(\frac{2\pi lB_it'}{N_i}-\frac{2\pi lm}{N_i}+\phi_l\right)\cdot\cos\left(\frac{2\pi vB_it'}{N_i}-\frac{2\pi vq}{N_i}+\phi_v\right)\right.\right.\right.\right.$$
$$\left.\left.\left.\left.+\sum_{l=1}^{\frac{N_i-1}{2}}\frac{2}{N_i^2}\cos\left(\frac{2\pi lB_it'}{N_i}-\frac{2\pi lm}{N_i}+\phi_l\right)+\sum_{l=1}^{\frac{N_i-1}{2}}\frac{2}{N_i^2}\cos\left(\frac{2\pi lB_it'}{N_i}-\frac{2\pi lq}{N_i}+\phi_l\right)+\frac{1}{N_i^2}\right)\right](f)\cdot\Pi\left(\frac{N_if}{B_i}\right)\right)\right](t)$$

$\overset{(4)}{=}$ is a consequence from applying a trigonometric product expansion formula.

$$\overset{(4)}{=}\left[\mathbf{F}_f^{-1}\left(\left[\mathbf{F}_{t'}\left(\sum_{v=1}^{\frac{N_i-1}{2}}\sum_{l=1}^{\frac{N_i-1}{2}}\left(\frac{2}{N_i^2}\cos\left(\frac{2\pi B_i(l+v)t'}{N_i}-\frac{2\pi lm}{N_i}-\frac{2\pi vq}{N_i}+\phi_l+\phi_v\right)\right.\right.\right.\right.\right.$$
$$\left.+\frac{2}{N_i^2}\cos\left(\frac{2\pi B_i(l-v)t'}{N_i}-\frac{2\pi lm}{N_i}+\frac{2\pi vq}{N_i}+\phi_l-\phi_v\right)\right)+\sum_{l=1}^{\frac{N_i-1}{2}}\frac{2}{N_i^2}\cos\left(\frac{2\pi lB_it'}{N_i}-\frac{2\pi lm}{N_i}+\phi_l\right)$$
$$\left.\left.\left.\left.+\sum_{l=1}^{\frac{N_i-1}{2}}\frac{2}{N_i^2}\cos\left(\frac{2\pi lB_it'}{N_i}-\frac{2\pi lq}{N_i}+\phi_l\right)+\frac{1}{N_i^2}\right)\right](f)\cdot\Pi\left(\frac{N_if}{B_i}\right)\right)\right](t)$$

$\overset{(5)}{=}$ follows by applying the filter and hence all t-dependent cosine components are removed, so that just the constant offset parts with a bandwidth in the filter range are still there and $\overset{(6)}{=}$ results from rewriting the orthogonality property using a N_i-line sinc sequence with a bandwidth of 1.

$$\overset{(5)}{=}\frac{1}{N_i^2}+\sum_{l=1}^{\frac{N_i-1}{2}}\frac{2}{N_i^2}\cos\left(-\frac{2\pi lm}{N_i}+\frac{2\pi lq}{N_i}\right)\overset{(6)}{=}\frac{1}{N_i}\mathrm{sq}_{N_i,1}(m-q)\,. \quad (7)$$

Finally, it can be seen, that the last expression of Eq. (7) corresponds to the right side of Eq. (5), where the orthogonality is expressed in a form of a sinc sequence. This means, that m and q also can be real-valued non-integers and the last expression of Eq. (7) gives the result for that generalized case.

References

1. Meier, J. *et al.* High-Bandwidth Arbitrary Signal Detection Using Low-Speed Electronics. *IEEE Photonics J.* **14**, 1 (2022).
2. Schmalen, L., van Wijngaarden, A. J. de L. & Ten Brink, S. Forward error correction in optical core and optical access networks. *Bell Labs Tech. J.* **18**, 39–66 (2013).
